# Outcomes of pregnancies that screened positive for sex chromosome aneuploidy ascertained via cell‐free DNA screening

**DOI:** 10.1002/jgc4.70107

**Published:** 2025-09-15

**Authors:** Cali FitzGerald, Shama P. Khan, Pranali Shingala, Gary Heiman, Elena Ashkinadze

**Affiliations:** ^1^ Genetic Counseling Master's Program Rutgers University Piscataway New Jersey USA; ^2^ Division of Genetic and Genomic Medicine, Department of Pediatrics UPMC Children's Hospital of Pittsburgh Pittsburgh Pennsylvania USA; ^3^ Division of Maternal Fetal Medicine, Department of Obstetrics and Gynecology Rutgers Robert Wood Johnson Medical School New Brunswick New Jersey USA; ^4^ Department of Genetics and the Human Genetics Institute of New Jersey Rutgers, the State University of New Jersey Piscataway New Jersey USA

**Keywords:** decision making, genetic counseling, genetic counselors, genetic testing, genetics services, prenatal diagnosis

## Abstract

Cell‐free DNA screening (cfDNA), also referred to as noninvasive prenatal screening (NIPS), is utilized to screen for fetal chromosomal aneuploidies during pregnancy, including sex chromosome aneuploidies (SCAs). All patients within our center are offered diagnostic testing following a positive cfDNA for an SCA, but not all patients pursue this testing. This retrospective chart review aims to improve understanding of how often patients undergo confirmatory diagnostic testing when cfDNA is positive or inconclusive for an SCA and the pregnancy outcomes, including pregnancy termination and live birth rates. We also describe the outcomes of cases where patients had a normal cfDNA result; however, the cfDNA‐predicted fetal sex is discrepant from the ultrasound‐predicted fetal sex. The study found that 56 patients had a positive or inconclusive cfDNA for SCA, and 36/56 (64.3%) pursued confirmatory testing either via prenatal (19 patients) or postnatal (17 patients) diagnostic testing. For the cases where confirmatory diagnostic information was available, an SCA was confirmed in 16/36 (44.4%). A birthing parent SCA was discovered to be the likely cause of a positive cfDNA in two cases. The positive predictive value (PPV) of cfDNA was 41.7% for all SCAs, 27.8% for Turner syndrome, 50.0% for triple X syndrome, 100% for Klinefelter syndrome, 100% for Jacobs syndrome, and 0% for inconclusive results. Nine patients had a negative cfDNA; however, the cfDNA‐predicted fetal chromosomal sex was discrepant from the fetal phenotypic sex predicted by ultrasound. In 3/9 cases, this led to a fetal ascertainment of a difference of sex development (DSD), which would not have been possible without the cfDNA result.

**What is known about this topic**

Research on the clinical utility, validity, and outcomes of screening for the most common autosomal trisomies (trisomies 21, 18, and 13) is abundant; however, there is less known about the impact of screening for SCAs. Previous studies have found that cfDNA for SCA is less specific than cfDNA for autosomal trisomies, leading to a higher rate of false positive results.

**What this paper adds to the topic**

To our knowledge, this is the largest study (nine cases) on fetal sex discrepancies between cfDNA and sonography. We also discuss considerations for post‐test counseling of patients who screen positive or inconclusive for an SCA and patients with identified fetal sex discrepancies.

## 1 INTRODUCTION

Cell‐free DNA screening (cfDNA) is a method used to detect fetal aneuploidies by analyzing the amount of fetal cell‐free DNA within the birthing parent circulation. This technology was first offered commercially in 2011 to detect the most common autosomal trisomies, 21, 18, and 13, which research has shown to be highly accurate (Bianchi & Wilkins‐Haug, 2014; Taylor‐Phillips et al., 2016). Beginning in 2012, cfDNA has expanded to include screening for sex chromosome aneuploidies (SCAs), also known as X and Y chromosome variations (Bianchi et al., 2015). SCAs are a group of disorders characterized by the gain or loss of one or more sex chromosomes and include Klinefelter syndrome (47, XXY), triple X syndrome (47, XXX), Turner syndrome (45, X), and Jacobs syndrome (47, XYY). The overall prevalence of SCAs is estimated to be around 1 in 440 newborns (Breman & Stankiewicz, 2021; Nielsen & Wohlert, 1991).

While research on the clinical utility, validity, and outcomes of screening for the most common autosomal trisomies is abundant, there is less known about the impact of screening for SCAs. It is essential to study the effects of screening for SCAs because their detection with cfDNA is not as specific as the detection of the common autosomal trisomies. SCAs also have a more variable phenotype, making it difficult to counsel patients on the implications of a positive result. Manifestations of an SCA may include decreased fertility, developmental delay, learning difficulties, and characteristic physical features; however, many individuals have no clinical findings (Skuse et al., 2018). Previous studies have shown that patients diagnosed with an SCA prenatally have improved health outcomes due to the early implementation of speech, physical, occupational, and hormonal therapies if needed (Bardsley et al., 2013; Linden & Bender, 2002; Ross et al., 2017; Samango‐Sprouse et al., 2019; Wigby et al., 2016). Most parents of children with an SCA agree that an early diagnosis had a positive impact on their child's life (Samango‐Sprouse et al., 2020). This proven benefit underlines the importance of researching the decisions patients make following a positive cfDNA for SCA.

With the introduction of cfDNA into clinical practice, the incidence of invasive procedures has declined while the diagnostic yield from these tests has increased (Awomolo et al., 2018). As the use of cfDNA for fetal genotypic sex determination has increased, there have been cases reported in which the cfDNA‐predicted fetal sex and the ultrasound‐predicted fetal sex are discrepant. Etiologies behind these discrepancies include sex chromosome aneuploidies of the fetus or the birthing parent, birthing parent organ transplant from a male donor, vanishing twin syndrome, differences of sex development (DSD), or human error by the laboratory or ultrasound technician (Byers et al., 2019; Dhamankar et al., 2020). Identifying a DSD prenatally has been shown to improve health outcomes in these individuals and may even change how a pregnancy is managed. For example, a diagnosis of congenital adrenal hyperplasia due to 21‐hydroxylase deficiency can be identified in 46, XX fetuses with virilization seen on prenatal ultrasound. A prenatal diagnosis can lead to prompt evaluation and treatment with hydrocortisone after birth to avoid a salt‐wasting crisis (Uslar et al., 2023). A prenatal DSD diagnosis also prevents delays in diagnosis later in life, as some individuals may only come to medical attention when experiencing delayed puberty or infertility. An earlier diagnosis allows for earlier implementation of hormone replacement therapy which has been shown to improve bone mineral density, cardiovascular health, sexual function, and general well‐being (Nordenström et al., 2018). Given these proven benefits of prenatal diagnosis for individuals with a DSD, we sought to study how the identification of a fetal sex discrepancy affects pregnancy management and outcomes. It is important to note that the stigmatization and discrimination of individuals with a DSD is well‐documented in the medical literature, but further research is needed to understand how prenatal genetic testing for these conditions impacts people with intersex variation (Zeeman & Aranda, 2020; Chapman & Benn, 2013).

In 2020, the American College of Obstetricians and Gynecologists (ACOG) issued a practice bulletin on screening for fetal chromosomal differences in which they recommend prenatal genetic screening and diagnostic testing be “discussed and offered to all pregnant patients regardless of age or risk for chromosomal abnormality” (Rose et al., 2020). As cfDNA is becoming more integrated into routine patient obstetrical care, it is important to study the pregnancy outcomes of individuals who screen positive for an SCA or fetal sex discrepancy to understand the impact of this screening.

In this study, we present data regarding prenatal diagnostic testing uptake rates, pregnancy termination rates, and postnatal testing uptake rates in patients whose cfDNA was high risk for an SCA. This includes patients whose cfDNA predicted an SCA and patients whose cfDNA‐predicted fetal sex that was discrepant with the ultrasound‐predicted fetal sex. We also examine demographic and clinical factors that may have influenced these decisions.

## 2 METHODS

### 2.1 Participants

A retrospective chart review was conducted of records from patients who had cfDNA screening at a single institution, the Division of Maternal Fetal Medicine, at Rutgers Robert Wood Johnson Medical School in New Brunswick, New Jersey, from January 1, 2013, to December 31, 2020. We chose this date range as 2013 was the first full year that cfDNA for SCA was offered at our institution. The cfDNA screening used at this institution included both massively parallel sequencing (MPS) and single nucleotide polymorphism (SNP) based testing, both of which reported on the most common types of SCA including Klinefelter syndrome, triple X syndrome, Turner syndrome, and Jacobs syndrome. More rare types of SCA such as tetrasomies (i.e., 48, XXYY) or pentasomies (i.e., XXXXY) may be reported on the cfDNA screening modalities utilized; however, they are not reported as reliably as the more common SCAs. All patients were consented for cfDNA by a genetic counselor, and all patients with an abnormal cfDNA had an appointment with a genetic counselor to discuss the result, either in person or telehealth. Eligible patients included all patients whose cfDNA revealed an increased risk for a fetal SCA, had a “no‐call” result for the sex chromosomes, or whose cfDNA‐predicted fetal sex was discrepant with the ultrasound‐predicted fetal sex. All data were de‐identified. This study was approved by the Rutgers University New Brunswick Health Sciences Institutional Review Board (IRB) and waived as human subjects research.

### 2.2 Instrumentation/procedures

Eligible subjects were identified via a clinical database that tracks all cfDNA results ordered through this practice. Once eligible patients were identified, their consult records and genetic test results were reviewed within the electronic medical record (EMR). The following data were abstracted from the EMR: indication for testing, patient and partner's demographics, gravida and parity, whether they had a history of infertility, mode of conception, need for translation services, insurance coverage, consultation type, cfDNA report, ultrasound findings, if the patient opted for further diagnostic testing and the result of this testing, and the outcome of the pregnancy. For pregnancies that resulted in a live birth, information on the health of the baby, including gestational age, birthweight, and whether postnatal genetic testing was coordinated, was abstracted. All information was collected and recorded in Microsoft Excel.

The patients were then separated into two groups. Group I included those with cfDNA results that were high risk for an SCA. Group II included patients with a cfDNA sex chromosome result that was typical but discrepant with the ultrasound‐predicted fetal sex. Patients who had an uninterpretable or no‐call sex chromosome cfDNA result were included in Group I due to the increased risk for chromosomal differences with no‐call results, especially for sex chromosomes (Gil et al., 2017; Rose et al., 2020).

### 2.3 Data analysis

Descriptive, unweighted analyses including frequencies, means, and standard deviations were used to describe patient characteristics and outcomes for subjects within Group I. We also calculated an indirect estimate of the PPV of cfDNA by dividing the number of true positive test results confirmed by prenatal or postnatal karyotype by the total number of positive screen results. To calculate the PPV for inconclusive results, we divided the number of cases with any SCA confirmed on prenatal or postnatal karyotype by the total number of inconclusive cfDNA sex chromosome results. Univariate analyses were conducted using Fisher's exact test and Stata statistical software (StataCorp, 2021).

Data for subjects within Group II were described, but statistical analyses were not completed due to the small sample size.

### 2.4 Bivariate analyses

After data collection, subjects in Group I were separated into two groups: patients who pursued prenatal diagnostic testing (cases) and patients who declined prenatal diagnostic testing (controls). After separating the data, we conducted association tests for each independent variable with the outcome variable. For categorical variables, we used Fisher's exact test. For continuous variables, we used logistic regression analysis.

## 3 RESULTS

### 3.1 Subject characteristics

**FIGURE 1:**
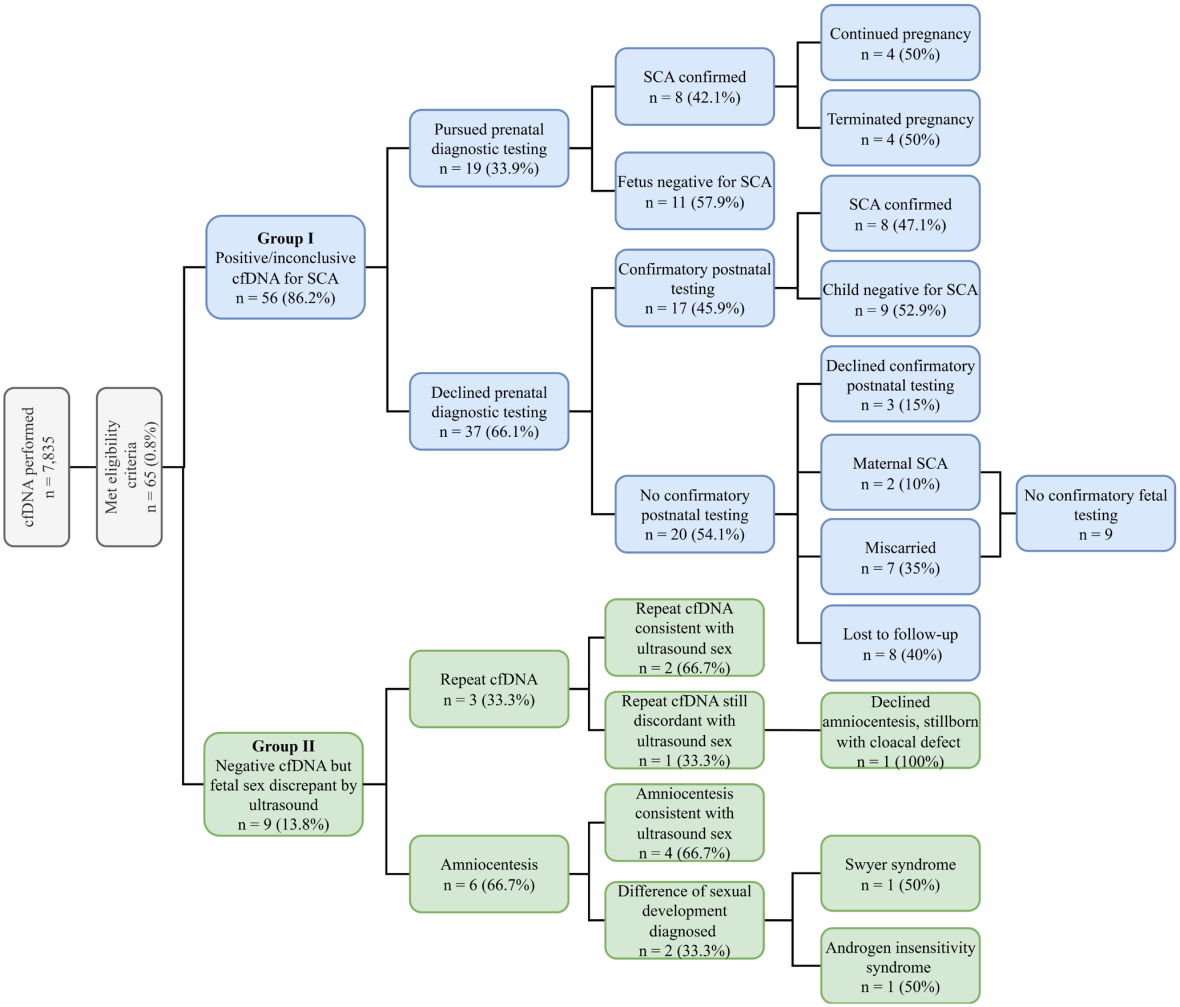
Overview of patient decisions and outcomes.

From January 1, 2013, to December 31, 2020, 7835 patients underwent cfDNA. A total of 65/7835 (0.8%) met eligibility criteria for the study. Group I had a total of 56 subjects. Confirmatory diagnostic testing was available for 36/56 (64.0%) subjects in Group I: 19/56 (34.0%) had invasive prenatal testing, and 17/56 (30.0%) had postnatal confirmatory testing. An SCA was confirmed in 16/36 (44.4%). In two cases, a birthing parent SCA, rather than a fetal SCA, was discovered (Figure 1).

Group II included nine subjects. Of the patients in Group II, 3/9 (33.3%) pursued a repeat cfDNA and 6/9 (66.7%) pursued an amniocentesis. In 6/9 (66.7%) patients, the repeat cfDNA or fetal karyotype via amniocentesis was concordant with the fetal genitalia observed by ultrasound. However, 3/9 (33.0%) fetuses were discovered to have a syndrome causing sex reversal (Figure 1).

Parental demographic and clinical information for all subjects (i.e., Group I and II) included in this study is summarized in Table 1. The mean age of study subjects was 34.6 ± 5.6 years with a range of 18–46 years old. The mean reproductive partner age was 35.3 ± 6.1 years with a range of 19–52 years old. The most common patient‐reported race/ethnicity was White (44.6%), followed by Hispanic (26.2%), Asian (16.9%), and Black (12.3%). White was also the most reported reproductive partner race/ethnicity (38.5%), followed by Hispanic (27.7%), Asian (16.9%), and Black (16.9%). For the majority of patients, the primary indication for cfDNA was advanced maternal age (AMA) (64.6%).

**TABLE 1:** Demographic and clinical characteristics for all subjects.

	*N* (%)
Birthing parent age	
<30	9 (13.9%)
30–34	19 (29.2%)
35–39	24 (36.9%)
≥40	13 (20.0%)
Birthing parent race/Ethnicity	
White	29 (44.6%)
Hispanic	17 (26.2%)
Asian	11 (16.9%)
Black	8 (12.3%)
Birthing parent highest level of education	
Post‐secondary	31 (73.8%)
High school	9 (21.4%)
Middle school	1 (2.4%)
Elementary	1 (2.4%)
Unspecified	23 (Not included)
Gravida	
Primigravida	15 (23.1%)
Multigravida	42 (64.6%)
Grand multigravida	8 (12.3%)
Number of living children	
Zero	27 (41.5%)
One	21 (32.3%)
Multiple	17 (26.2%)
Indication for cfDNA	
AMA	42 (64.6%)
Routine screening	14 (21.5%)
Other abnormal screen	5 (7.7%)
Ultrasound finding	4 (6.2%)
Mode of conception	
Artificial reproductive technology (ART)	3 (4.6%)
Spontaneous	62 (95.4%)
Personal history of infertility	
Yes	7 (10.8%)
No	58 (89.2%)
Translation services utilized	
Yes	6 (9.2%)
No	59 (90.8%)
Health insurance	
Public or uninsured	14 (21.5%)
Private	51 (90.8%)
Reproductive partner age	
<30	7 (11.5%)
30–34	17 (27.9%)
35–39	26 (42.6%)
≥40	11 (18.0%)
Unspecified	4 (Not included)
Reproductive partner race/Ethnicity	
White	25 (38.5%)
Hispanic	18 (27.7%)
Asian	11 (16.9%)
Black	11 (16.9%)
Reproductive partner highest level of education	
Post‐secondary	19 (61.3%)
High school	11 (35.5%)
Middle school	1 (3.2%)
Unspecified	34 (Not included)

### 3.2 Group I results

Of the 56 patients who had an increased risk for an SCA based on cfDNA, 25 (44.6%) were for Turner syndrome, 15 (26.8%) were for triple X syndrome, seven (12.5%) were for Klinefelter syndrome, four (7.1%) were for Jacobs syndrome, and five (8.9%) had an inconclusive sex chromosome result.

#### 3.2.1 Patients in group I who pursued prenatal diagnostic testing

Nineteen (33.9%) patients with positive cfDNA for an SCA decided to pursue prenatal diagnostic testing (6 CVS, 13 amniocentesis), and a total of four (42.1%) were confirmed as true positives. Based on prenatal diagnostic results, 4/8 (50.0%) patients decided to terminate their pregnancy. Of the patients who terminated their pregnancy, one fetus had Turner syndrome with a cystic hygroma on ultrasound, two fetuses had triple X syndrome with no ultrasound findings, and one fetus had an increased risk for Klinefelter syndrome with cfDNA and a 48, XXYY karyotype on amniocentesis with no ultrasound findings (Table 2).

**TABLE 2:** Prenatal diagnostic testing uptake, diagnostic yield, and termination rates.

cfNDA result	Prenatal diagnostic testing uptake rate	Diagnostic yield	Termination rate
Turner Syndrome (45, X)	40.0% (10/25)	10.0% (1/10)	100.0% (1/1)
Triple X Syndrome (47, XXX)	26.7% (4/15)	75.0% (3/4)	66.7% (2/3)
Klinefelter Syndrome (47, XXY)	42.9% (3/7)	100.0% (3/3)^a^	33.3% (1/3)^b^
Jacobs Syndrome (47, XYY)	25.0% (1/4)	100.0% (1/1)	0.0% (0/1)
Inconclusive	20.0% (1/5)	0.0% (0/1)	0.0% (0/0)
Total	33.9% (19/56)	42.1% (8/19)	50.0% (4/8)

^a^ One fetus had a 48, XXYY karyotype.

^b^ This pregnancy was the fetus with a 48, XXYY karyotype.

An abnormal ultrasound finding was seen in 13/56 (23.0%) Group I subjects (Table 3). Nine of these findings are associated with the indicated SCA, including cystic hygroma, increased nuchal translucency (NT), cardiac anomalies, renal anomalies, small for gestational age (SGA), and intrauterine growth restriction (IUGR). Of the patients with ultrasound findings, 4/13 (30.8%) pursued prenatal diagnostic testing, of which 3/4 (75.0%) had findings associated with the SCA. Half of the patients with ultrasound findings who pursued prenatal diagnostic testing were confirmed as true positives. There were 43/56 (76.8%) patients in Group I with no ultrasound findings, and 15/43 (34.9%) pursued prenatal diagnostic testing. The presence of ultrasound findings did not increase the chance that a patient would pursue prenatal diagnostic testing compared to patients without ultrasound findings (30.8% vs. 34.9%, respectively), though our sample size was small at only 13 cases with ultrasound findings.

**TABLE 3:** Ultrasound findings.

cfDNA result	Without US finding	With US finding	Percent with US finding	Ultrasound findings				
				Finding	Count (*n*)			
					True positive	False positive	SAB or LFU	Total
Turner Syndrome (45, X)	17	8	32.0% (8/25)	Cystic hygroma	1	–	2	3
				Increased NT	–	1	–	1
				EIF	–	1	–	1
				IUGR	2	–	–	2
				Pyelectasis & IUGR	1	–	–	1
Triple X Syndrome (47, XXX)	12	3	20.0% (3/15)	Abnl head shape	–	1	–	1
				Absent nasal bone	1	–	–	1
				VSD, DOLV	1	–	–	1
Klinefelter Syndrome (47, XXY)	7	0	0.0% (0/7)	–	–	–		
Jacobs Syndrome (47, XYY)	2	2	50.0% (2/4)	Bilateral choroid plexus cysts	1	–	–	1
				IUGR	–	–	1	1
Inconclusive	5	0	0.0% (0/5)	–	–	–		
Total	43	13	23.2% (13/56)	–	7	3	3	13

Abbreviations: abnl, abnormal; DOLV, double outlet left ventricle; EIF, echogenic intracardiac focus; IUGR, intrauterine growth restriction; LFU, lost to follow‐up; NT, nuchal translucency; SAB, spontaneous abortion; US, ultrasound; VSD, ventricular septal defect.

#### 3.2.2 Patients in group I who declined prenatal diagnostic testing

37/56 (66.1%) patients with an increased risk for SCA did not pursue prenatal diagnostic testing. Seven patients did not pursue prenatal diagnostic testing because of a spontaneous miscarriage. Two of the pregnancies that ended in a spontaneous miscarriage had screened high risk for Turner syndrome with a cystic hygroma visualized on ultrasound, four pregnancies screened high risk for Turner syndrome without ultrasound findings or ultrasound not yet performed, and one pregnancy had uninterpretable sex chromosomes. Of the patients who had a spontaneous miscarriage, one patient chose to pursue chromosome analysis on products of conception (POC), however the culture failed.

Two patients whose cfDNA was high risk for triple X syndrome did not pursue prenatal diagnostic testing because birthing parent chromosome analysis revealed a 47, XXX karyotype, indicating their abnormal cfDNA result was birthing parent in origin.

The remaining 28 patients who screened high risk for SCA on cfDNA and did not pursue prenatal diagnostic testing gave birth to a liveborn infant. Eight patients were lost to follow‐up. Three patients (one triple X syndrome, two Klinefelter syndrome) chose not to pursue postnatal diagnostic testing, while 17 patients pursued postnatal diagnostic testing. The karyotypes in these cases revealed 10 newborns had typical sex chromosomes (46, XY (1), 46, XX (9)). Of these 10 newborns with typical postnatal karyotypes, four had received a positive cfDNA for Turner syndrome, four for triple X syndrome, and two with inconclusive sex chromosomes. Seven newborns had an SCA diagnosed postnatally, summarized in Table 4.

**TABLE 4:** Postnatally confirmed SCA outcomes.

Subject ID	cfDNA result	Postnatal karyotype	Postnatal outcome
6	45, X	40% 45, X, 60% 47, XXX in 100 cells counted	Born FT, weighed 5 lb. 12 oz. Microcephaly noted at birth. Normal ECHO and ECG. No other health problems noted at birth
12	47, XXX	47, XXX	Born FT, weighed 6 lb. 3 oz. Non‐dysmorphic, no health problems noted at birth
13	45, X	45, X	Born FT, weighed 5 lb. 9 oz. Admitted to NICU for respiratory distress. Broad chest with widely spaced nipples and cubitus valgus noted on physical exam. ECHO revealed mild coarctation of the aorta and partial anomalous pulmonary venous return
24	45, X	46, X, del(X)(p22.33)	Born FT, weighed 5 lb. 5 oz. Widely spaced nipples and bilateral low‐set ears noted on physical exam
26	45, X	45, X/46, XX	Born at a different hospital. Further postnatal health outcomes unknown
31	47, XYY	47, XYY	Born at a different hospital. Further postnatal health outcomes unknown
39	47, XXX	47, XXX	Born FT, weighed 6 lb. 3 oz. Non‐dysmorphic, no health problems noted at birth

Abbreviations: ECG, electrocardiogram; ECHO, echocardiogram; FT, full‐term.

There was a total of 36 patients who pursued follow‐up testing, either prenatally or postnatally, after receiving a positive cfDNA for an SCA. The PPV of cfDNA was as follows: 41.7% (15/36) for all SCAs, 27.8% (5/18) for Turner syndrome, 50.0% (5/10) for triple X syndrome, 100% (3/3) for Klinefelter syndrome, 100% (2/2) for Jacobs syndrome, and 0% (0/3) for inconclusive results. None of the demographic and clinical characteristic variables for Group I patients were statistically different between those who pursued and declined prenatal diagnostic testing (Table S1).

### 3.3 Group II results

Nine patients had a cfDNA‐predicted fetal sex that was discrepant from the ultrasound‐predicted fetal sex, summarized in Table S2. After a fetal sex discrepancy was identified, all patients were informed of the option to repeat cfDNA with a different laboratory or have an amniocentesis. Repeat cfDNA or amniocentesis was consistent with the ultrasound‐predicted fetal sex in 6/9 cases (66.7%).

A DSD was confirmed or suspected in 3/9 (33.0%) subjects in Group II. One fetus, part of a dizygotic twin pregnancy, was predicted to be genotypically male by cfDNA and phenotypically female by ultrasound. The patient underwent a medically indicated preterm delivery at 22 weeks. One fetus had male genitalia, and the other fetus had a cloacal defect with ambiguous genitalia. Birthing parent androgen insensitivity syndrome (AIS) carrier screening was negative, and no molecular testing was done on the fetus. The patient declined further testing. Two other fetuses were predicted to be genotypically male by cfDNA and phenotypically female by ultrasound. Both pregnancies underwent molecular testing via amniocentesis, and one fetus was confirmed to have a pathogenic hemizygous SRY exon 1 deletion consistent with Swyer syndrome, while the other was positive for a hemizygous novel pathogenic variant (c.2461G>T, p.G821W) in the *AR* gene consistent with AIS.

## 4 DISCUSSION

### 4.1 Diagnostic testing uptake

One goal of our study was to understand how often patients undergo confirmatory diagnostic testing when cfDNA is positive for an SCA and what factors may impact a patient's decision to pursue diagnostic testing. Most patients (64.3%) pursued confirmatory diagnostic testing. However, only 19/56 (33.9%) of patients in our study opted for prenatal confirmatory testing. This small prenatal diagnostic testing uptake rate may be due to patients knowing they would not change their pregnancy management based on an SCA diagnosis. This is reflected in the high termination rate (50.0%) in patients in our study who received a prenatally confirmed SCA, a rate that is consistent with prior reports of 55.6% by So et al. (2017) and 53.8% by Yao et al. (2014) Prior research has also shown that individuals who would consider pregnancy termination with a positive diagnostic test were more likely to undergo invasive testing (Di Mattei et al., 2021).

Within Group I, none of the variables we studied were found to be significantly associated with pursuing prenatal diagnostic testing (Table S1). This may be due to the small sample size. Another explanation may be that there are other variables that affect a patient's decision to pursue prenatal diagnostic testing that were not included in our study. While we collected information on previous abnormal prenatal screens such as nuchal translucency and serum screening, we did not record results from normal prenatal screening or carrier screening. Prior research has shown that there are many additional factors that influence a patient's decision to pursue prenatal diagnostic testing which could not be obtained in this retrospective study. This includes ethical and religious beliefs, acceptability and perceived risk of having a child with an SCA, views on pregnancy termination, parental anxiety level, tolerance of uncertainty, and level of genetic literacy (Cheng, Chen, & Wang, 2018; Di Mattei et al., 2021; Marteau, et al., 1991; Pivetti, Melotti, Morselli, & Olivieri, 2013; Wilson, Ferguson, & Thorn, 2011).

### 4.2 Performance of cfDNA for SCA

Previous studies have shown that cfDNA for SCA is less specific than cfDNA for autosomal trisomies, leading to a higher rate of false positive results (Kornman et al., 2018). In our study, cases at a high risk for SCA with cfDNA where prenatal or postnatal confirmatory testing results were available showed a PPV of 41.7% for all SCAs. This is consistent with previous studies which found the combined PPV of cfDNA for SCAs to range from 32.4% to 57.6% with an average of 43.8% (Deng et al., 2019; Lu et al., 2021; Reiss et al., 2017; Wang et al., 2020). In our study, the PPV for Turner syndrome was 27.8%. This is comparable to previous studies which found the PPV for Turner syndrome to be considerably lower than all other SCAs at 20.0% (Bianchi & Wilkins‐Haug, 2014) and 21.4% (Wang et al., 2020). The PPV for triple X syndrome was 50.0%. All cases of positive cfDNA for Klinefelter and Jacobs syndromes with outcome data were true positives (PPV = 100%). The sample size was small at three cases of Klinefelter syndrome and two cases of Jacobs syndrome; however, this high PPV is consistent with other studies which found the PPV for Klinefelter syndrome to be 90.0% and 75.0% for Jacobs syndrome (Wang et al., 2020). None of the cases of inconclusive cfDNA for the sex chromosomes with available outcome data were true positives; however, the sample size was small at only three cases.

### 4.3 Impact of utilizing cfDNA for SCA screening

Since sex chromosome cfDNA screening was introduced in 2012, there have been concerns regarding the ethics, utility, and psychosocial impact of such testing. Research has identified concerns for the future child's right to genetic privacy, the impact on the future child's self‐esteem, and the stigmatization that individuals with an SCA or DSD face (Dondorp et al., 2015; Hens, 2018; Johnston et al., 2022; Zeeman & Aranda, 2020). There have also been concerns raised regarding the clinical utility of cfDNA screening for SCA detection as the phenotypic variability of SCAs makes it difficult to predict the future health outcomes for the fetus (Johnston et al., 2022).

Despite these challenges, research has shown an improvement in neurodevelopmental outcomes for patients diagnosed with an SCA prenatally as they are able to begin therapies at an earlier age, if needed (Bardsley et al., 2013; Linden & Bender, 2002; Ross et al., 2017; Samango‐Sprouse et al., 2019; Wigby et al., 2016). Prenatal diagnosis has also been shown to be beneficial for parents because it allows them to research the condition before the birth of their child, connect with support groups, and meet with the doctors who will be providing care for their child (Samango‐Sprouse et al., 2020). While there are both pros and cons to prenatal screening for SCAs, it is important to ensure that parents are offered the choice to pursue diagnostic testing, both prenatally and postnatally, after a positive cfDNA screen to maintain patient autonomy throughout the process.

### 4.4 Practice implications

Multiple professional societies, including the American College of Medical Genetics and Genomics (ACMG), ACOG, and the International Society for Prenatal Diagnosis (ISPD), have published practice guidelines regarding cfDNA screening for prenatal aneuploidy assessment (Dungan et al., 2023; Hui et al., 2023; Rose et al., 2020). These practice guidelines provide some guidance for pre‐ and post‐test counseling of patients with positive cfDNA results for SCA; however, they do not address cases of fetal sex discrepancies. For example, ACOG recommends completing invasive diagnostic testing in patients with a screen‐positive cfDNA test; however, they do not address recommendations for fetal sex discrepancy cases (Rose et al., 2020). We encourage professional societies to provide clear guidance related to the care of patients with an identified fetal sex discrepancy. Below, we outline the information we believe would be critical to consider in the development of these guidelines.

If genotypic sex prediction is included in the fetal cfDNA report, the sonographer and/or obstetrician performing the fetal level II ultrasound should confirm that the ultrasound‐predicted fetal sex is consistent with the cfDNA‐predicted fetal sex. This is especially important in cases where the parents chose not to know the fetal sex, as this may be the only opportunity to discover a fetal DSD. Providers may consider offering their patients with a fetal sex discrepancy invasive diagnostic testing including direct fetal karyotype, microarray analysis, and/or a DSD workup. For patients that decline a prenatal workup and the cfDNA‐predicted fetal sex is discrepant from the ultrasound‐predicted fetal sex, providers may consider arranging testing on tissue (if a pregnancy loss) or the newborn immediately after birth. A diagnosis is important for medical management and recurrence risk counseling for future pregnancies.

### 4.5 Study limitations

One limitation of our study was that we did not have postnatal testing on 20/56 (35.7%) subjects. In some cases, we know that testing was offered but not completed. However, in most cases, we do not have any additional information because the patients were lost to follow‐up. Therefore, we cannot draw any conclusions about these patients. A future study should follow a prospective approach, and follow‐up for patients should be standardized to truly measure the parameters discussed in our study. Another limitation of our study was the small sample size and that all patients were seen at a single institution, making it difficult to generalize our results to a broader population.

## AUTHOR CONTRIBUTIONS

Cali FitzGerald, Elena Ashkinadze, Pranali Shingala, Shama P. Khan, and Gary Heiman each fulfilled the International Committee of Medical Journal Editors criteria for authorship. Cali FitzGerald contributed to the conceptualization, data curation, formal analysis/data interpretation, investigation, methodology creation, project administration, and identification of resources for this study. She wrote the original draft and actively reviewed and revised additional drafts. Elena Ashkinadze contributed to the conceptualization, data curation, formal analysis/data interpretation, investigation, methodology creation, project administration, and supervision for this study. She assisted in writing the original draft and reviewed and revised additional drafts. Pranali Shingala contributed to the data curation, project administration, and supervision of this study in addition to reviewing and revising drafts. Shama P. Khan contributed to the conceptualization, data curation, project administration, and supervision of this study in addition to reviewing and revising drafts. Gary Heiman participated in the data curation, formal analysis/data interpretation, methodology creation, project administration, and supervision of this study in addition to reviewing and revising drafts. Authors Cali FitzGerald and Elena Ashkinadze confirm that they had full access to all the data in the study and take responsibility for the integrity of the data and the accuracy of the data analysis. All of the authors gave final approval of this version to be published and agree to be accountable for all aspects of the work in ensuring that questions related to the accuracy or integrity of any part of the work are appropriately investigated and resolved.

## CONFLICT OF INTEREST STATEMENT

Cali FitzGerald, Shama P. Khan, Pranali Shingala, Gary Heiman, and Elena Ashkinadze declare that they have no conflicts of interest.

## Supporting information


Appendix S1.

## Data Availability

The data that support the findings of this study are available from the corresponding author upon reasonable request.
