# Supplementary material for: Outcomes of pregnancies that screened positive for sex chromosome aneuploidy ascertained via cell‐free DNA screening
Source: J Genet Couns. 2025 Sep 15;34(5):e70107. doi: 10.1002/jgc4.70107 (PMC12435101; doi:10.1002/jgc4.70107)
Supplement: Supplementary file 1 — Appendix S1. [file JGC4-34-0-s001.docx]

**Appendix**

*Supplementary Table 1: Demographic and clinical characteristics of Group I patients who pursued and declined prenatal diagnostic testing.*

| **Characteristic** | **Declined Prenatal Diagnostic Testing, n** | **Pursued Prenatal Diagnostic Testing, n** | ***p*-value** |
| --- | --- | --- | --- |
| **Total** | 37 | 19 |  |
| **Indication for cfDNA** |  |  |  |
| AMA | 22 | 14 | 0.38 |
| Other | 15 | 5 |  |
| **Birthing parent age, mean (SD)** | 34.6 (6.5) | 35.4 (3.1) | 0.61 |
| **Birthing parent race/ethnicity** |  |  |  |
| White | 16 | 7 | 0.19 |
| Hispanic | 12 | 4 |  |
| Black | 6 | 2 |  |
| Asian | 3 | 6 |  |
| **Reproductive partner race/ethnicity** |  |  |  |
| White | 14 | 6 | 0.51 |
| Hispanic | 12 | 4 |  |
| Black | 7 | 4 |  |
| Asian | 4 | 5 |  |
| **Parental marital status** |  |  |  |
| Unmarried | 7 | 3 | 1.0 |
| Married | 28 | 16 |  |
| **Gestational age in days, mean (SD)** | 95.4 (29.0) | 90.3 (14.8) | 0.48 |
| **Gravida** |  |  |  |
| Primigravida | 8 | 4 | 1.0 |
| Multigravida | 17 | 9 |  |
| Grand multigravida | 12 | 6 |  |
| **Number of living children** |  |  |  |
| Zero | 13 | 9 | 0.64 |
| One | 12 | 6 |  |
| Multiple | 12 | 4 |  |
| **Infertility** |  |  |  |
| No | 33 | 17 | 1.0 |
| Yes | 4 | 2 |  |
| **>2 miscarriages** |  |  |  |
| No | 31 | 15 | 0.72 |
| Yes | 6 | 4 |  |
| **Insurance type** |  |  |  |
| Public or uninsured | 9 | 5 | 0.87 |
| Private | 28 | 14 |  |
| **cfDNA predicted fetal sex** |  |  |  |
| Female | 26 | 14 | 1.0 |
| Male | 8 | 4 |  |
| Inconclusive | 3 | 1 |  |
| **cfDNA result** |  |  |  |
| Turner | 15 | 10 | 0.70 |
| Trisomy (trisomy X, - - Klinefelter, Jacobs) | 18 | 8 |  |
| Inconclusive | 4 | 1 |  |
| **Abnormal ultrasound** |  |  |  |
| No | 28 | 15 | 1.0 |
| Yes | 9 | 4 |  |

*Supplementary Table 2: Outcomes of pregnancies with discrepant cfDNA and ultrasound predicted fetal sex.*

| **Subject ID** | **cfDNA Predicted Sex (FF)** | **Ultrasound Predicted Sex** | **Outcome** |
| --- | --- | --- | --- |
| 55 | Male (10.0%) | Female | Opted for repeat cfDNAwith another company. cfDNA consistent with 46,XX female. |
| 56 | Female (8.0%) | Male | Opted for amnio. Karyotype: 46,XY. |
| 59 – dizygotic twin pregnancy | Male/Male (3.6%/3.4%) | Male/Female | Developed hemolysis, elevated liver enzymes, low platelet count (HELLP) syndrome, medically indicated preterm delivery at 22 weeks. One fetus had male genitalia and the other fetus had genitourinary anomalies including ambiguous genitalia. Birthing parent AIS carrier screening negative. Pt declined further testing. |
| 60 | Male (10.0%) | Female | Opted for amnio. Karyotype: 46,XX female. |
| 61 | Female (6.5%) | Male | Opted for repeat cfDNA with another company. cfDNA consistent with 46,XY male. |
| 62 | Male (14.0%) | Female | Opted for amnio. Karyotype: 46,XX female. |
| 63 | Male (3.4%) | Female | Opted for amnio. Karyotype: 46,XX female. |
| 64 | Male (14.0%) | Female | Opted for amnio at 17 weeks 6 days: FISH probes for SRY and DXZ1 showed normal 46,XY male, aCGH microarray normal 46,XY male. 3D ultrasound at 18 weeks 2 days showed more male appearing genitalia with microphallus and possible hypospadias. Repeat amnio was performed and a 46,XY Disorders of Sex Development panel with 19 genes was ordered. Positive for a pathogenic hemizygous SRY exon 1 deletion consistent with Swyer syndrome. |
| 65 – di/di twin pregnancy | Y material detected, unable to determine if one or both fetuses are male | Female/Female | Pregnancy conceived via IVF with PGD for aneuploidy, embryos transferred predicted to be 46,XX and 46,XY. After the 16 week ultrasound showed female genitalia for both fetuses, pt pursued cfDNA. Amnio was performed at 20 weeks. Karyotypes: 46,XX (twin A) and 46,XY (twin B). SNP microarray normal for both twins. SRY FISH analysis for twin B was normal. Androgen Insensitivity Syndrome testing on twin B was positive for a hemizygous novel pathogenic variant (c.2461 G>T, p.G821W) in the *AR* gene. Pt pursued carrier testing for the variant and was positive. |
| FF = fetal fraction, pt = patient, FISH = fluorescence in situ hybridization | | | |
